# Effect of the Helium Background Gas Pressure on the Structural and Optoelectronic Properties of Pulsed-Laser-Deposited PbS Thin Films

**DOI:** 10.3390/nano11051254

**Published:** 2021-05-11

**Authors:** Ameni Rebhi, Anouar Hajjaji, Joël Leblanc-Lavoie, Salma Aouida, Mounir Gaidi, Brahim Bessais, My Ali El Khakani

**Affiliations:** 1 Photovoltaic Laboratory, Research and Technology Centre of Energy (CRTEn), BP 95, 2050 Hammam-Lif, Tunisia; amenrabhi@gmail.com (A.R.); physicshajjaji@gmail.com (A.H.); salma.aouida@crten.rnrt.tn (S.A.); mkaidi@sharjah.ac.ae (M.G.); brahim.bessais@crten.rnrt.tn (B.B.); 2 Centre Énergie Matériaux et Télécommunications (INRS-EMT), Institut National de la Recherche Scientifique (INRS), 1650 Boulevard Lionel Boulet, Varennes, QC J3X 1S2, Canada; joel.leblanc-lavoie@inrs.ca; 3 Faculty of Sciences, University of Tunis El Manar, 2092 Tunis, Tunisia; 4 Center of Advanced Research Materials, Research Institute of Sciences and Engineering, University of Sharjah, Sharjah P.O. Box 27272, United Arab Emirates

**Keywords:** pulsed laser deposition (PLD), PbS thin films, background pressure effect, morphology, bandgap, electrical resistivity, optoelectronic properties

## Abstract

This work focuses on the dependence of the features of PbS films deposited by pulsed laser deposition (PLD) subsequent to the variation of the background pressure of helium (P_He_). The morphology of the PLD-PbS films changes from a densely packed and almost featureless structure to a columnar and porous one as the He pressure increases. The average crystallite size related to the (111) preferred orientation increases up to 20 nm for P_He_ ≥ 300 mTorr. The (111) lattice parameter continuously decreases with increasing P_He_ values and stabilizes at P_He_ ≥ 300 mTorr. A downshift transition of the Raman peak of the main phonon (1LO) occurs from P_He_ = 300 mTorr. This transition would result from electron–LO–phonon interaction and from a lattice contraction. The optical bandgap of the films increases from 1.4 to 1.85 eV as P_He_ increases from 50 to 500 mTorr. The electrical resistivity of PLD-PbS is increased with P_He_ and reached its maximum value of 20 Ω·cm at P_He_ = 300 mTorr (400 times higher than 50 mTorr), which is probably due to the increasing porosity of the films. P_He_ = 300 mTorr is pointed out as a transitional pressure for the structural and optoelectronic properties of PLD-PbS films.

## 1. Introduction

Lead sulfide (PbS) continues to attract interest because of its unique optoelectronic properties and associated applications. It is a semiconductor having a narrow bulk direct bandgap of 0.41 eV at room temperature [1] and a large exciton Bohr radius of 18 nm [2]. It exhibits strong quantum confinement effects when synthesized under the form of nanoparticles (NPs) having an average size smaller than 18 nm. Consequently, the bandgap of PbS-NPs can be increased by simply decreasing their size. This latter aspect has been particularly exploited for the development of novel optoelectronic and photovoltaic PV devices [3,4]. In addition to the nanoparticle form, PbS thin films is another functional form of PbS that is highly interesting for various applications, including thin film solar cells [5,6] or infrared detectors [7,8,9]. In fact, the capacity of PbS films to efficiently absorb and convert light over a wide spectrum (ranging from UV, to visible to near-IR), and their long exciton lifetime (0.2–0.8 ms) are major assets that make them very suitable for various optoelectronic device applications. Thus, by varying the deposition methods and their associated operating conditions, it is possible to optimize the optoelectronic properties of the PbS films in response to a specific application [10,11,12,13]. Most often, PbS thin films have been deposited by various chemical techniques, including Chemical Bath Deposition (CBD) [14], Successive Ionic Layer Adsorption and Reaction (SILAR) [15], electrodeposition [16], and spray pyrolysis method [17]. The popularity of chemical deposition techniques is due to their low cost and ease of implementation even if the final quality of the PbS films is not always optimal and the optoelectronic performance of their associated devices is poor [18,19]. On the other hand, physical vapor deposition (PVD) techniques (including sputtering [20,21], thermal evaporation [22], atomic layer deposition [23], or pulsed laser deposition (PLD) [24,25,26]) have been used to deposit PbS thin films onto different substrates. Among these PVD methods, PLD is widely used to deposit high-quality thin films [27,28,29] onto various substrates. One of the primary advantages of PLD is its ability to preserve the stoichiometry of complex materials when deposited from a multi-element target [30,31]. The highly energetic aspect of the ablated species (up to 10 times higher than in sputtering) is another unique feature of the PLD process that enables the growth of crystalline phases even at room temperature (RT) [31]. Finally, the properties of PLD-thin films strongly depend on the deposition parameters such as laser fluence, number of laser ablation pulses (NLP), gas pressure, deposition time, and temperature [31,32]. For instance, the effect of substrate temperature and argon background pressure has been investigated by Beatriceveena et al. [26] in the case of PbS films deposited by PLD onto (012) LaAlO_3_ substrates. Thus, they have identified an optimal deposition temperature and pressure (553 K and 75 mTorr, respectively) for the growth of PbS films having an average (200) crystallite size of 25 nm and a resistivity of 5 kΩ·cm. The effect of deposition temperature was also studied by Dhlamini et al. [33] in the case of PLD-PbS films embedded into an amorphous silica host. They have shown that the increase of the deposition temperature from room temperature to 400 °C increases the particle size in PbS-films and their PL intensity, which was found to consistently redshift with deposition temperature. Other work has highlighted using moderate laser intensities since high laser flux densities have been reported to cause degradation in the structural quality of PbS films and an increase of their surface roughness [34]. Despite these various studies on the PLD deposition parameters, no systematic study has been reported yet on the effect of the in situ background pressure of inert gas on the structure and properties of pulsed-laser-deposited PbS films. For example, it is known that the PLD deposition rate decreases as the background gas pressure increases (as a consequence of a higher confinement of the laser ablation plume in the background gas) because of the reduced mean free path of the ablated species when the gas pressure is increased [35]. This would definitely influence the growth mechanism, the morphology, and hence the properties of the PLD-PbS films. An investigation that is still to come, particularly in the case of inert background gas (He), is one that enables singling out the physical effects while avoiding the added complexity of chemical ones resulting from a reactive gas.

In this paper, we report on a systematic study of the effect of the He background pressure (P_He_) on the structural, morphological, and optoelectronic properties of the PbS films deposited using the PLD technique. Thus, by varying the residual pressure inside the PLD deposition chamber over a wide range (from 5 × 10^−5^ to 0.5 Torr), we were able to point out significant changes in the crystallinity, morphology, and optoelectronic properties of the PbS films. A helium (He) background pressure of 300 mTorr was identified as a tipping point at which the deposition rate, crystallite size, and electrical resistivity reached their maximum values. In particular, it is shown that the electrical resistivity of the PLD-PbS films can be adjusted from 0.05 to 20 Ω·cm when the P_He_ is raised from 50 to 300 mTorr, while their associated optical bandgap increases from 1.4 to 1.65 eV. The electrical resistivity behavior with P_He_ can be interpreted in terms of the P_He_-induced porosity change of the PLD-PbS films.

## 2. Materials and Methods

The PLD of PbS films was performed by using a KrF excimer laser (wavelength = 248 nm, pulse duration = 14 ns, repetition rate = 20 Hz, and pulse energy = 120 mJ), which was focused on a rotating PbS pellet at an incident angle of 45°. Each deposition was simultaneously made on (100)-oriented Intrinsic-Si and quartz substrates at room temperature. These substrates were placed on a rotating 3″-diameter substrate holder, which is placed parallel to the target at a distance of 7.5 cm. Prior to their loading on the substrate holder, the substrates were degreased by acetone, cleaned with isopropanol and de-ionized water, and then dried under a dry nitrogen (N_2_) jet. The PLD depositions were made under vacuum (≈5 × 10^−5^ Torr) and under different He background pressures ranging from 50 to 500 mTorr of He. Before each PbS film deposition, the PLD chamber was first pumped with a dry pump (to reach ≈50 mTorr residual pressure), turbo-pumped down to ≈5 × 10^−5^ Torr, and then filled with He gas up to the desired working pressure. The morphological, structural, electrical, and optoelectronic properties of the PbS thin films were systematically characterized as a function of PHe. The films investigated here were deposited with a fixed number of laser ablation pulses (N_LP_) of 3500. Prior to each deposition, the target surface was in situ cleaned by ablating its surface for 5 min while appropriately protecting the substrate holder from the laser ablation plume by a shutter placed close to the target.

The thickness and morphology of the PLD-PbS films were systematically examined using scanning electron microscopy (SEM). Their crystalline structure was characterized, over the 2θ range 20–55°, by using an X’Pert Pro X-ray diffractometer employing a CuKα (λ = 1.5418 Å) radiation, at a grazing incidence angle of 1.5°. The UV-Vis optical transmission of the films was determined using a Perkin Elmer Lambda 750 UV-visible-NIR spectrophotometer (in the wavelength range of 200–1500 nm). Raman spectra were acquired using a Renishaw Raman spectrometer using an Argon-ion laser (λ = 532 nm) at a fixed power of 10 mW (the incident laser was focused with a spot of ≈2 µm-diameter on the samples). The Fourier transform infrared (FTIR) spectroscopy was used to characterize the nature of the chemical bondings of the PLD-PbS films by means of a Nicolet 6700 FTIR spectrometer. The resistivity of the PLD-PbS films as a function of P_He_ was systematically determined by carrying out room temperature four-point probes electrical measurements. Ohmic contacts were formed on the four corners of the samples using small gold blobs.

## 3. Results and Discussion

**Figure 1:**
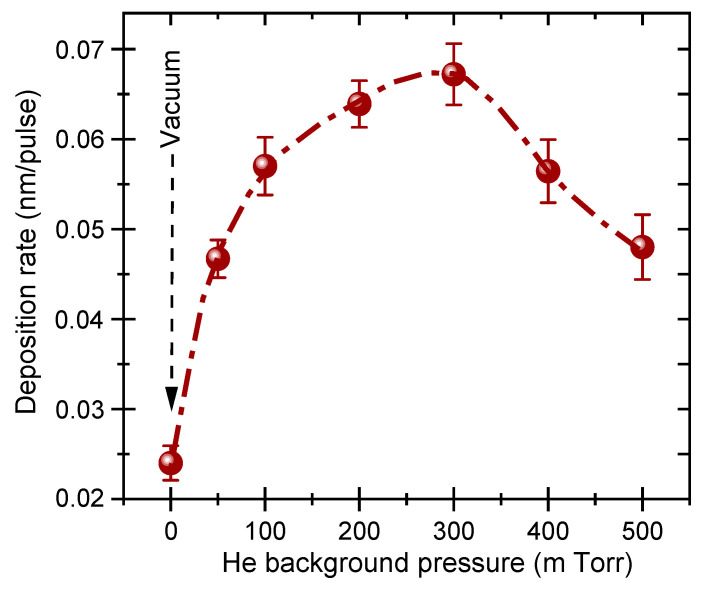
Helium background pressure dependence of the deposition rate of PbS films.

Figure 1 shows the He pressure dependence of the deposition rate (D_rt_) of the PLD-PbS films. It is clearly seen that D_rt_ increases with P_He_ and reaches a maximum (0.067 nm/pulse) at P_He_ = 300 mTorr, a pressure after which D_rt_ continues to drop until it reaches ≈0.047 nm/pulse at 500 mTorr. This clearly points to P_He_ = 300 mTorr as the optimal condition that leads to the thickest films. This increase in the apparent thickness of the PbS films is a consequence of an increase of their porosity, as it has been previously reported for both IrO_2_ and SnO_2_ deposited by PLD under background gas pressures in the same range as in the present study [36,37,38]. In fact, under vacuum and low He pressures, the ablated species are highly energetic and contribute to the growth of densely packed films. As the pressure is increased, the ablated species lose part of their kinetic energy through collisions with background gas molecules and “smoothly land” on the substrate with less energy, which limits their surface diffusivity and favors the growth of progressively porous structures. This phenomenon is likely what dominates the growth of PbS films until P_He_ is increased up to 300 mTorr, where a maximum of the ablated species can still reach the substrate even if they are significantly slowed down. At higher P_He_ (≥400 mTorr), the mean free path of the ablated species is significantly reduced, resulting in a stronger confinement of the ablated species close to the target, and an increasing proportion of the ablated species cannot reach the substrate any more. This marks the decline of the deposition rate, even if the porosity of the films can still increase (as this will be shown hereafter in the SEM images at 500 mTorr). Thus, the maximum D_rt_ observed at P_He_ = 300 mTorr is a sort of trade-off between two opposite phenomena: on one hand, the slowing down of the ablated species, which tends to increase the apparent thickness of the films because of the increase of their porosity and, on the other hand, the progressive diminution of the mean-free path of the ablated species, which limits the amount of matter that can reach the substrate. Ultimately, if P_He_ is further increased, the confinement of the ablation plume will be so effective that most of the ablated species will condense in the gas phase and fall down near the target, and no film will be deposited on the substrate that is placed 7.5 cm away (then, D_rt_ will be close to zero). The confinement of the laser ablation plume can be visually seen (through the visible luminescence part of the excited species of the plasma) in the deposition chamber through a viewport. In fact, as the background pressure is increased from vacuum to 500 mTorr of He, the shape of the ablation plume significantly changes from a slender elongated plume (under vacuum or very low P_He_) to an almost pseudo-spherical ball confined close to the target, reflecting somehow the significant reduction in the mean free path of the ablated species. The effect of increasing background gas pressures on the shape and dynamics of nanosecond laser ablation plumes has been well documented [31,39,40].

**Figure 2:**
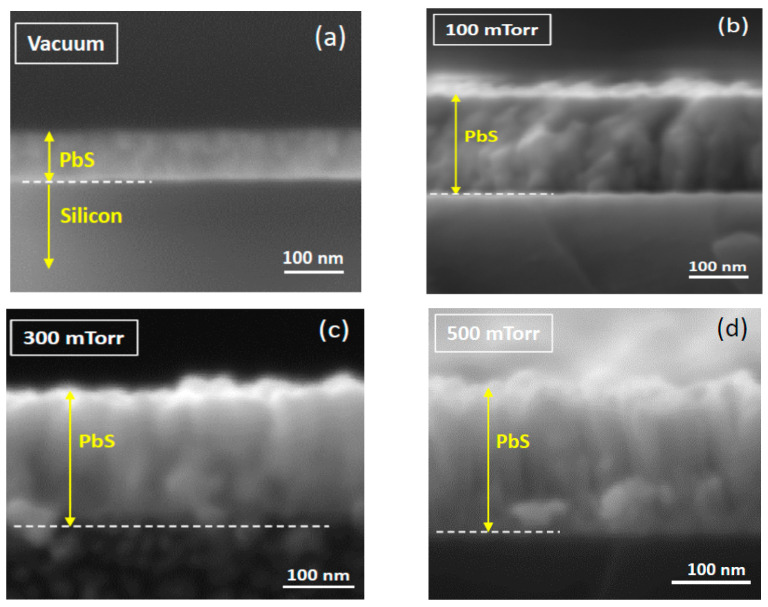
Typical SEM images of the PbS films deposited at N_LP_ = 3500, under vacuum (**a**) and (**b**–**d**) different He background pressures.

Figure 2 shows cross-section SEM images of the PLD-PbS films deposited on silicon substrates under different He pressures. It is clearly seen that the PbS films deposited under vacuum (Figure 2a) are densely packed with an almost featureless morphology. As P_He_ is increased, the morphology of the films develops toward a more porous and less dense packed one (Figure 2b–d). At 500 mTorr, loosely packed PbS features (columnar-like) are clearly seen to present an intercolumnar porosity. These observations corroborate well with the above-described P_He_ dependence of D_rt_ in the sense that under vacuum, the energetic ablated species form a very dense (hammered) PbS films, while at P_He_ = 500 mTorr (for example), the soft landing of the PbS ablated species (with much less energetic budget) leads to the formation of a more porous (fluffy) films. At the optimal value of P_He_ = 300 mTorr (that gives the highest D_rt_), the PLD-PbS films are seen to exhibit an in-between morphology, which can be described as a good mix between relatively dense columnar morphology and a certain level of porosity.

**Figure 3:**
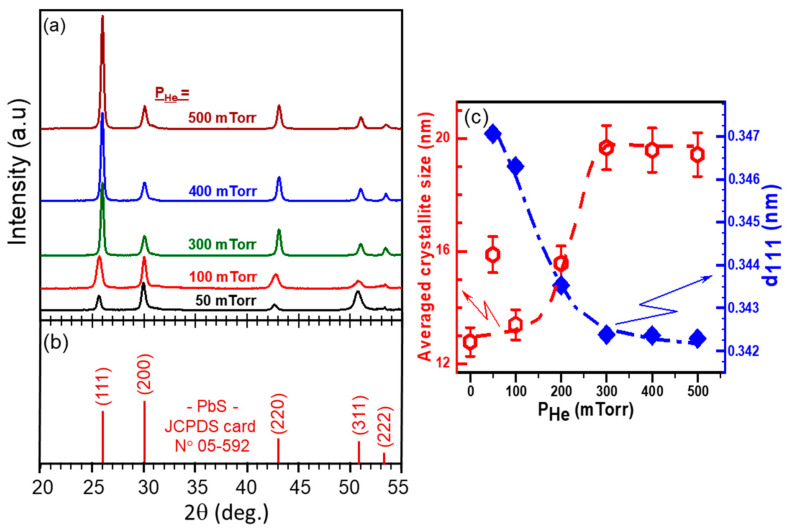
(**a**) XRD patterns of PbS films deposited at different He pressures ranging from 50 to 500 mTorr (at N_LP_ = 3500); (**b**) Standard XRD peak positions and intensities for PbS powder according to the JCPDS card N° 5-592); and (**c**) He pressure dependence of both the crystallite size and lattice parameter of the (111) plans (d111).

Figure 3a shows the XRD patterns of PLD-PbS films deposited on Si substrates under various He pressures. For comparison purposes, the standard XRD peaks of PbS powder with its associated crystallographic plans (JCPDS card N° 05-592) are also depicted in Figure 3b. Two main observations can be made: first, all the XRD peaks exhibited by the PLD-PbS films are in agreement with the fingerprint of PbS, confirming the polycrystalline nature of the PbS films even if they were PLD-deposited at room temperature; secondly, one can notice that the PLD-PbS films have exhibited a preferential (111) orientation. Both observations are rather consistent with the unique features of PLD. In fact, the highly energetic aspect of the laser-ablated species provides a higher surface diffusivity to atoms, which would favor the crystallization of PbS without any additional heating of the substrate. On the other hand, PLD is known for its unequalled very-high instantaneous deposition rate, which favors the in-plane growth of the densest (111) crystallographic plans. Finally, no others peaks associated with PbO, PbO_2_, PbSO_3_, PbSO_4_, etc. were observed, confirming thereby the pure PbS cubic phase of our films. To analyze further the XRD peaks, we have calculated the interplanar distance d_111_ and the averaged crystallite size from the majority crystallographic orientation (111). Figure 3c shows the P_He_ dependence of both crystallographic parameters. The interplanar distance d_111_ is found to drop markedly when P_He_ is increased up to 300 mTorr and then stabilizes for higher P_He_ values. On the other hand, the average (111) crystallite size shows almost an opposite variation as a function of P_He_. It increases from ≈13 nm to ≈20 nm when the background pressure is varied from vacuum to 300 mTorr; then, it stabilizes around 20 nm for higher P_He_ values. Similar grain-size increase with increasing background gas pressures has been previously reported for both PLD-deposited SnO_2_, silicon, and titanium silicate films [37,41,42]. The increase of crystallite size observed here can be understood when considering the amount of ablated matter that reaches the substrate and the slowing down of the ablated species as the P_He_ is increased (as reflected from the P_He_ dependence of D_rt_ shown in Figure 1). In fact, as P_He_ is increased up to 300 mTorr, the kinetic energy of the ablated species decreases, but they can still condense on the substrate and contribute to the growth of existing nucleated nanocrystals (likely not enough energy to diffuse on the substrate and create new nucleation sites). This is consistent with the observed increase of PbS crystallites up to ≈20 nm. Beyond P_He_ = 300 mtorr, the residual energy of the ablated species continues to diminish, and a decreasing amount of the ablated matter can still make it to condense onto the substrate surface. This limits the growth of the nanocrystals, of which average size is seen to not grow larger than ≈20 nm. Concomitantly, the highly supersaturated flux of ablated species that land on the growing film with a decreasing surface diffusivity (as P_He_ is increased) leads to a very rapid condensation of a mechanically stressed PbS film. Such stress is expected to be mostly in the out-of-plane direction of the film (the direction of arrival of ablated species) where less relaxation and diffusion is permitted. The overall stress field sustained by the film is believed to cause the observed lattice parameter contraction (Figure 3c), particularly when P_He_ is increased from 50 to 300 mTorr, which is the same pressure range where the deposition rate significantly increases and reaches its maximum at 300 mTorr (Figure 1). It is worth recalling here that the magnitude of the in-plane compressive stress component, which is induced by the “hammering” of the growing film by the energetic ablated species, is expected to diminish as P_He_ is increased (slowing down of the ablated species).

**Figure 4:**
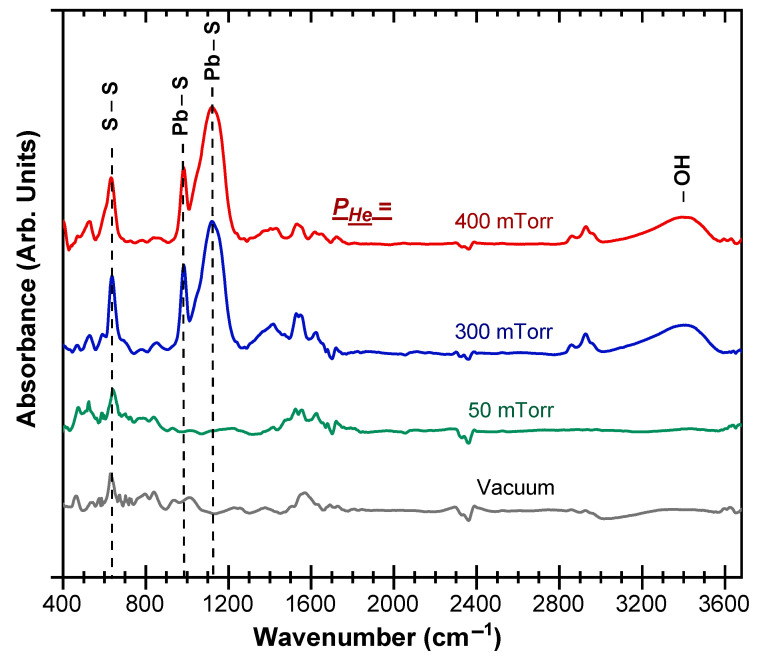
FTIR spectra of the PbS films pulsed-laser-deposited on silicon substrates under vacuum and selected He background pressures (50, 300, and 400 mTorr).

The FTIR spectroscopy has been also used to characterize the bonding and local environments of the PLD-PbS films as a function of P_He_. Figure 4 shows the FTIR spectra of PLD-PbS films deposited onto intrinsic and double-side-polished silicon substrates, under vacuum and selected He background pressures (50, 300, and 400 mTorr). Since all the films were deposited at a fixed N_LP_ of 3500, their thicknesses are ≈85, ≈150, ≈240, and 200 nm for vacuum, 50, 300, and 400 mTorr of P_He_, respectively. All the spectra exhibit a sharp FTIR peak located at 630 nm, which is due to the S-S stretching vibrations [43,44]. This peak is relatively intense even in the very thin films because of the high oscillator strength of the S-S stretching vibrations. Two other prominent peaks located at 980 and 1122 cm^−1^ are characteristic of PbS [45]. These FTIR peaks are consistent with the crystalline PbS pure phase characterized by XRD. One can also note the presence of a broad band (around 3411 cm^−1^) that is more prominent for thicker and more porous films.

This band is due to the OH-stretching vibrations of hydrogen-bonded hydroxyl groups. The presence of such hydroxyl groups is due the adsorption of water molecules (from ambient humidity) by the accessible surface of PbS films. Finally, the absence of Pb-S peaks (at 980 and 1122 cm^−1^) for the films deposited under vacuum and 50 mTorr of He is due to the rather limited sensitivity of the FTIR as those films have thicknesses of ≈150 nm or less. Indeed, XRD results (see Figure 3a above) have clearly confirmed the presence of the polycrystalline PbS phase for all the investigated background pressures (even if the signal is also relatively low for thinner films deposited under vacuum or 50 mTorr of He).

**Figure 5:**
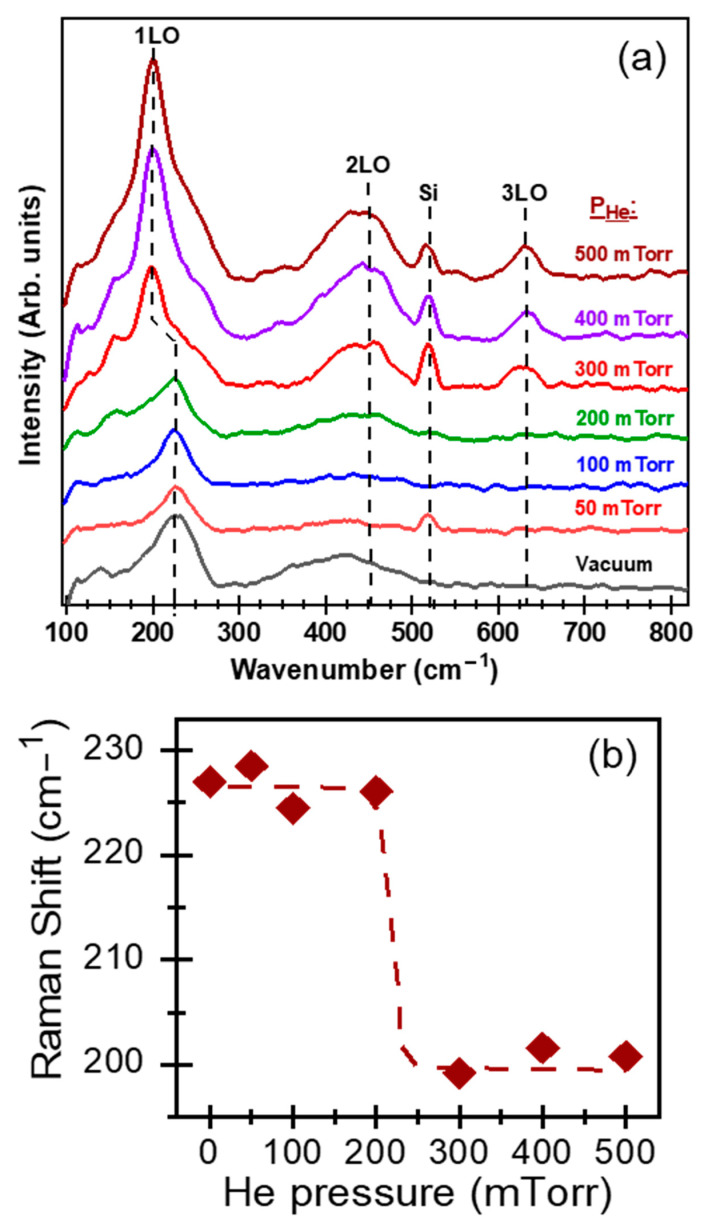
(**a**) Raman spectra of the PLD PbS films deposited on silicon substrates under different background pressures (at N_LP_ = 3500); (**b**) Position of the 1LO phonon peak as a function of helium pressure.

The Raman spectra of the PLD-PbS films (Figure 5a) revealed several resolved bands. The broad bands peaking at 200 cm^−1^, 437 cm^−1^, and 630 cm^−1^ are related to the first, second, and third harmonics of the longitudinal optical (LO) phonon mode, respectively [46,47]. The line peaking at 520 cm^−1^ corresponds to the first-order optical phonon of the underlying silicon substrate [48]. By fitting the 1LO Raman peak by a Lorentzian line, we determined its full width at half maximum (FWHM), which is found to reach the lowest value of ≈45 cm^−1^ (for P_He_ ≥ 300 mTorr), indicating thereby the improved crystallinity of the films (in good agreement with the above-discussed XRD results; Figure 3c). On the other hand, the position of the 1LO dominant Raman peak is seen to remain unchanged (around 227 cm^−1^) over all the P_He_ pressure range of 50–200 mTorr range and abruptly shifts to ≈200 cm^−1^ for P_He_ ≥ 300 mTorr (Figure 5b). This significant transition in the 1LO Raman peak (shift toward smaller wavenumbers) is highly likely due to the observed abrupt increase of the (111) crystallite size from 13 to 20 nm for P_He_ ≥ 300 mTorr (red curve in Figure 3c). Similar downshifting of the 1LO position Raman peak has been previously reported when the PbS crystallite size increased from ≈17 to ≈36 nm [49]. In fact, such an 1LO peak shift has been associated with the increase of PbS nanocrystals, as a consequence of a decreased electron–LO–phonon interaction in the nanocrystals as they grow in size [50]. Moreover, one can note also that the observed downshifting of the 1LO peak correlates well with the (111) lattice parameter contraction that was revealed to take place for P_He_ ≥ 300 mTorr (blue curve of Figure 3c). A similar behavior has been reported for ZnS nanowires, where a large surface tension is present [51]. In the case of PLD-PbS films, lattice parameter contraction can be due to strong in-plane mechanical stress sustained by the films, particularly by the (111) densest crystallographic PbS plans. Finally, another plausible contribution to the observed 1LO-peak downshifting could be due to the presence of adsorbed –OH groups in the PbS porous structure (as revealed by FTIR measurements for P_He_ ≥ 300 mTorr; see Figure 4). This has been highlighted for PbSe nanocrystals of which the 1LO Raman peak was shown to downshift when the films were exposed to air [52].

**Figure 6:**
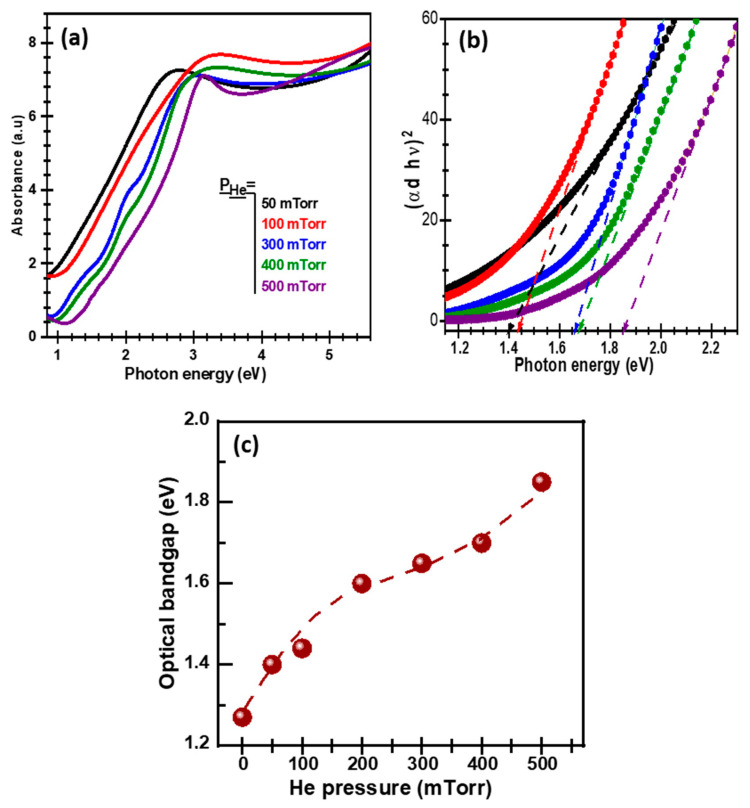
(**a**) UV-Vis absorbance spectra of PbS films deposited (at N_LP_ = 3500) onto quartz substrates at different helium background pressures. (**b**) Associated Tauc-plots from which the optical bandgap values were derived; (**c**) Variation of the optical bandgap of PLD-PbS films with background He pressure.

Figure 6a depicts the absorbance spectra of PLD-PbS films deposited on quartz under increasing He background pressure. The absorption edge is found to blueshift continuously as the P_He_ is increased from 50 to 500 mTorr, indicating a clear change in the optoelectronic properties of the PbS films. To extract the optical bandgap (E_g_) of the PLD-PbS films, the UV-Vis absorbance spectra were transformed in their corresponding Tauc plots (Figure 6b), according to the following equation: (αt hν)^2^ = A (E_g_ − hν), where E_g_ is the optical bandgap, A is a constant, α is the absorption coefficient, h is the Plank constant, ν is the photon frequency, and t is the thickness of the film [53]. Figure 6c summarizes the P_He_ dependence of the Eg of PLD-PbS films. It is clearly shown that the optical bandgap energy of PLD-PbS film increases almost linearly from 1.3 eV (under vacuum) to 1.85 eV (under 500 mTorr of He), with a sort of a plateau (around 1.63 eV) between 200 and 300 mTorr of He. Even if the reasons of such bandgap widening (as a function of P_He_) are not completely understood and might be of different origins, one could invoke its possible correlation with the progressive decrease observed in the (111) lattice parameter (Figure 3c). In fact, the more the valence electrons become linked by decreasing the interatomic distance, the more energy is needed to make them free in the conduction band (which translates into wider bandgap). On the other hand, the Eg values obtained here are much larger than that quoted for bulk PbS (≈0.41 eV) [54]. In fact, we have previously reported large E_g_ values for PLD-PbS nanoparticles (of which size is smaller than the 18 nm of the exciton Bohr radius of PbS) as a consequence of quantum confinement (QC) occurrence [24]. Here, because the PLD-PbS films (85–250 nm thick) are much thicker than a single layer of PbS-NPs, large PbS-NPs form from the coalescence of smaller ones. Hence, it is difficult to invoke a direct QC induced bandgap widening, particularly for P_He_ ≥ 300 mTorr where the crystallite size was estimated to be of ≈20 nm, which is larger than the PbS exciton Bohr radius (keeping also in mind that a single nanoparticle can be formed by more than one crystallite). Nevertheless, even if the QC cannot be invoked in the actual PLD-PbS films, the PbS crystallites can still undergo spatial confinement (SC) that would enlarge their bandgap. Indeed, SC effects can be experienced if the size of the PbS crystallite is larger than the exciton Bohr radius but still relatively small (much smaller than the mean diffusion length of charge carriers in the bulk material) to restrict severely the diffusion of electrons, confining them spatially and decreasing strongly their probability to recombine at non-radiative sites. Such SC has been particularly reported in the case of silicon nanostructures where intense photoluminescence was observed at energies much higher than that of the bandgap of the bulk material even if the size of the Si nanocrystals is larger than the Bohr radius of Si (≈5 nm) [55,56].

**Figure 7:**
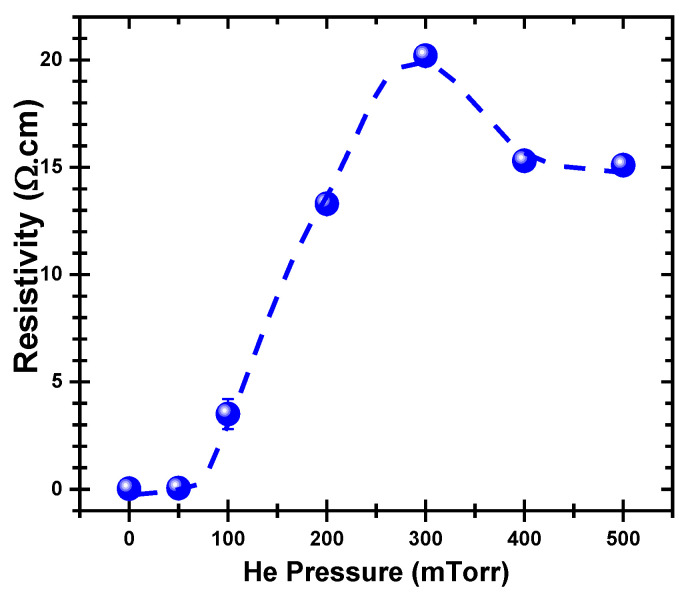
Variation of the electrical resistivity of the PLD-PbS films as a function of the He background pressure.

Finally, the electrical resistivity of the PLD-PbS films were measured as a function of P_He_ and reported in Figure 7. The resistivity of the PbS films is seen to remain almost unchanged when the films are deposited under vacuum or under 50 mTorr. When P_He_ is increased from 50 to 300 mTorr, the resistivity increases progressively from 0.05 to 20 Ω·cm. For higher pressures (P_He_ > 300 mTorr), the resistivity of the PbS films starts to drop and reaches ≈15 Ω·cm at P_He_ = 500 mTorr.

Figure 7 shows again that P_He_ = 300 mTorr is a tipping point, where the resistivity reaches its maximum. In fact, this resistivity increase (between 50 and 300 mTorr) is likely due to the increasing porosity of the films, which will hinder the charge carrier motion (increased scattering between PbS grains). For higher P_He_ (400 and 500 mTorr), the relative decrease of the resistivity could result from the increased adsorption of hydroxyl groups in the films (as revealed by FTIR). This being said, all the resistivity range (0.05 to 15 and to 20 Ω·cm) exhibited by our PLD-PbS films remains significantly lower than the values reported for spray-pyrolysis deposited PbS films, for example, for which the resistivity values were in the 145 to 1800 Ω·cm range for film thicknesses in the 520 down to 150 nm, respectively [57]. This shows that despite the relative porosity discussed above, the PLD-PbS films remain highly pure, densely packed, and more conductive in comparison with their chemically synthesized counterparts.

## 4. Conclusions

The pulsed laser deposition technique was successfully used to deposit PbS films under different He background pressures, affecting thereby their structure, morphology, and optoelectronic properties. By achieving systematic characterizations, we were thus able to highlight the changes in the PbS films’ microstructure, which is shown to change with P_He_ from highly dense and featureless morphology to a more porous and loosely packed columnar-like structure. These morphological changes were reflected into the P_He_ dependence of deposition rate, which reaches its maximum at 300 mTorr. All the PbS films are polycrystalline with a (111) preferential orientation. The (111) crystallite size was found to increase from ≈13 to 20 nm when P_He_ is raised up to 300 mTorr. All our results pointed to significant changes and/or transitional behaviors occurring around the 300 mTorr He pressure. This is believed to correspond to the PLD condition where the ablated species are sufficiently slowed down but can still reach the substrate with sufficient residual kinetic energy to grow large PbS crystallites (≈20 nm). Beyond this pressure, the amount of ablated matter that reaches the substrate starts to diminish and reduces the deposition rate. On the other hand, while the optical bandgap of the PLD-PbS films was found to increase from 1.4 to 1.85 eV (as P_He_ is increased from 50 to 500 mTorr), their electrical resistivity consistently increased with P_He_ (from 0.05 to 15–20 Ω·cm). These bandgap values are definitely much larger than that of bulk PbS (≈0.4 eV), but quantum confinement induced bandgap widening could not be simply invoked here. It is believed that the observed bandgap values could be due to different and/or a mixture of complex phenomena including spatial confinement, lattice parameter contraction, and/or surface defects states.

## References

[1] Madelung O. (2004). Semiconductors: Data Handbook.

[2] Touati B., Gassoumi A., AlFaify S., Kamoun-Turki N. (2015). Optical, morphological and electrical studies of Zn: PbS thin films. Mater. Sci. Semicond. Process..

[3] Sukhovatkin V., Hinds S., Brzozowski L., Sargent E.H. (2009). Colloidal Quantum-Dot Photodetectors Exploiting Multiexciton Generation. Science.

[4] Maraghechi P., Labelle A.J., Kirmani A.R., Lan X., Adachi M.M., Thon S.M., Sargent E.H. (2013). The Donor–Supply Electrode Enhances Performance in Colloidal Quantum Dot Solar Cells. ACS Nano.

[5] Rosario S.R., Kulandaisamy I., Arulanantham A.M.S., Kumar K.D.A., Valanarasu S., Shkir M., AlFaify S. (2019). Fabrication and characterization of lead sulfide (PbS) thin film based heterostructure (FTO/CdS/PbS/Ag) solar cell by nebulizer spray method. Mater. Res. Express.

[6] Moon D.G., Rehan S., Yeon D.H., Lee S.M., Park S.J., Ahn S., Cho Y.S. (2019). A review on binary metal sulfide heterojunction solar cells. Sol. Energy Mater. Sol. Cells.

[7] Liu X., Zhang M. (2000). Studies on PbS and PbSe Detectors for IR System. Int. J. Infrared Millim. Waves.

[8] Safrani T., Kumar T.A., Klebanov M., Arad-Vosk N., Beach R., Sa’Ar A., Golan Y. (2014). Chemically deposited PbS thin film photo-conducting layers for optically addressed spatial light modulators. J. Mater. Chem. C.

[9] Cheragizade M., Yousefi R., Jamali-Sheini F., Mahmoudian M.R., Sa A., Huang N.M. (2014). Synthesis and characterization of PbS mesostructures as an IR detector grown by hydrogen-assisted thermal evaporation. Mater. Sci. Semicond. Process..

[10] Aadim K., Ibrahim A., Marie J. (2017). Structural and optical properties of PbS thin films deposited by pulsed laser deposited (PLD) technique at different annealing temperature. Int. J. Phy..

[11] Barrios-Salgado E., Rodríguez-Lazcano Y., Pérez-Orozco J.P., Colin J., Altuzar P., Campos J., Quesada D. (2019). Effect of Deposition Time on the Optoelectronics Properties of PbS Thin Films Obtained by Microwave-Assisted Chemical Bath Deposition. Adv. Condens. Matter Phys..

[12] Göde F., Güneri E., Emen F.M., Kafadar V.E., Ünlü S. (2014). Synthesis, structural, optical, electrical and thermoluminescence properties of chemically deposited PbS thin films. J. Lumin..

[13] Bai R., Kumar D., Chaudhary S., Pandya D.K. (2017). Highly crystalline p-PbS thin films with tunable optical and hole transport parameters by chemical bath deposition. Acta Mater..

[14] Seghaier S., Kamoun N., Brini R., Amara A.B. (2006). Structural and optical properties of PbS thin films deposited by chemical bath deposition. Mater. Chem. Phys..

[15] Kanniainen T., Lindroos S., Ihanus J., Leskelä M. (1996). Growth of strongly orientated lead sulfide thin films by successive ionic layer adsorption and reaction (SILAR) technique. J. Mater. Chem..

[16] Alanyalıoğlu M., Bayrakçeken F., Demir Ü. (2009). Preparation of PbS thin films: A new electrochemical route for underpotential deposition. Electrochim. Acta.

[17] Thiagarajan R., Beevi M.M., Anusuya M., Ramesh T. (2012). Influence of reactant concentration on nano crystalline PbS thin films prepared by Chemical Spray Pyrolysis. Optoelectro. Adv. Mater. Rapid Commun..

[18] Sukharevska N., Bederak D., Goossens V.M., Momand J., Duim H., Dirin D.N., Loi M.A. (2021). Scalable PbS Quantum Dot Solar Cell Production by Blade Coating from Stable Inks. ACS Appl. Mater. Interfaces.

[19] Rohom A.B., Londhe P.U., Jadhav P.R., Bhand G.R., Chaure N.B. (2017). Studies on chemically synthesized PbS thin films for IR detector application. J. Mater. Sci. Mater. Electron..

[20] Guozheng M., Binshi X., Haidou W., Shuying C., Zhiguo X. (2014). Excellent Vacuum Tribological Properties of Pb/PbS Film Deposited by Rf Magnetron Sputtering and Ion Sulfurizing. ACS Appl. Mater. Interfaces.

[21] Da Silva Filho J.M.C., Marques F.C. (2019). Structural and optical temperature-dependent properties of PbS thin films deposited by radio frequency sputtering. Mater. Sci. Semicond. Process..

[22] Kumar S., Sharma T.P., Zulfequar M., Husain M. (2003). Characterization of vacuum evaporated PbS thin films. Phys. B Condens. Matter.

[23] Dasgupta N.P., Lee W., Prinz F.B. (2009). Atomic Layer Deposition of Lead Sulfide Thin Films for Quantum Confinement. Chem. Mater..

[24] Ka I., Ma D., El Khakani M.A. (2011). Tailoring the photoluminescence of PbS-nanoparticles layers deposited by means of the pulsed laser ablation technique. J. Nanopart. Res..

[25] Atwa D.M.M., Azzouz I.M., Badr Y. (2011). Optical, structural and optoelectronic properties of pulsed laser deposition PbS thin film. Appl. Phys. B.

[26] Beatriceveena T.V., Prabhu E., Jayaraman V., Gnanasekar K.I. (2019). X-ray photoelectron and Hall studies on nanostructured thin films of PbS grown by pulsed laser deposition. Mater. Lett..

[27] El Khakani M.A., Chaker M. (1998). Reactive pulsed laser deposition of iridium oxide thin films. Thin Solid Films.

[28] Ka I., Le Borgne V., Ma D., El Khakani M.A. (2012). Pulsed Laser Ablation based Direct Synthesis of Single-Wall Carbon Nanotube/PbS Quantum Dot Nanohybrids Exhibiting Strong, Spectrally Wide and Fast Photoresponse. Adv. Mater..

[29] Ka I., Gonfa B., Le Borgne V., Ma D., El Khakani M.A. (2014). Pulsed Laser Ablation Based Synthesis of PbS-Quantum Dots-Decorated One-Dimensional Nanostructures and Their Direct Integration into Highly Efficient Nanohybrid Heterojunction-Based Solar Cells. Adv. Funct. Mater..

[30] Gaidi M., Hajjaji A., Smirani R., Bessaïs B., El Khakani M.A. (2010). Structure and photoluminescence of ultrathin films of SnO_2_ nanoparticles synthesized by means of pulsed laser deposition. J. Appl. Phys..

[31] Chrisey D.B., Hubler G.K. (1994). Pulsed Laser Deposition of Thin Films.

[32] Popescu A.C., Stan G.E., Duta L., Nita C., Popescu C., Surdu V.A., Craciun V. (2015). The Role of Ambient Gas and Pressure on the Structuring of Hard Diamond-Like Carbon Films Synthesized by Pulsed Laser Deposition. Materials.

[33] Dhlamini M.S., Terblans J.J., Ntwaeaborwa O.M., Ngaruiya J.M., Hillie K.T., Botha J.R., Swart H.C. (2008). Photoluminescence properties of powder and pulsed laser-deposited PbS nanoparticles in SiO_2_. J. Lumin..

[34] Kumar S., Chandra R. (2009). Influence of laser flux density on the surface morphology of lead sulphide thin films. Chalcogenide Lett..

[35] Chen T., Li X.M., Zhang S., Zeng H.R. (2004). Oxygen-pressure dependence of the crystallinity of MgO films grown on Si(100) by PLD. J. Cryst. Growth.

[36] Riabinina D., Chaker M., Rosei F. (2006). Correlation between plasma dynamics and porosity of Ge films synthesized by pulsed laser deposition. Appl. Phys. Lett..

[37] Dolbec R., El Khakani M.A., Serventi A.M., Saint-Jacques R.G. (2003). Influence of the nanostructural characteristics on the gas sensing properties of pulsed laser deposited tin oxide thin films. Sens. Actuators B Chem..

[38] Hamoudi Z., El Khakani M.A., Mohamedi M. (2012). Influence of Pressure on the Structural and Electrocatalytic Properties of Pt Nanoparticles Grown by Pulsed Laser Ablation onto Carbon Paper Substrate. Int. J. Electrochem. Sci..

[39] Geohegan D.B., Puretzky A.A. (1995). Dynamics of laser ablation plume penetration through low pressure background gases. Appl. Phys. Lett..

[40] Farid N., Harilal S.S., Ding H., Hassanein A. (2014). Emission features and expansion dynamics of nanosecond laser ablation plumes at different ambient pressures. J. Appl. Phys..

[41] Kabashin A.V., Sylvestre J.P., Patskovsky S., Meunier M. (2002). Correlation between photoluminescence properties and morphology of laser-ablated Si/SiO_x_ nanostructured. J. Appl. Phys..

[42] Brassard D., El Khakani M.A. (2005). Pulsed-laser deposition of high-k titanium silicate thin films. J. Appl. Phys..

[43] Bakshi M.S., Thakur P., Sachar S., Kaur G., Banipal T.S., Possmayer F., Petersen N.O. (2007). Aqueous Phase Surfactant Selective Shape Controlled Synthesis of Lead Sulfide Nanocrystals. J. Phys. Chem. C.

[44] Nafees M., Ikram M., Ali S. (2017). Thermal stability of lead sulfide and lead oxide nano-crystalline materials. Appl. Nanosci..

[45] Giribabu K., Suresh R., Manigandan R., Vijayalakshmi L., Stephen A., Narayanan V. (2012). Hydrothermal Synthesis of Lead Sulphide Nanoparticles and their Electrochemical Sensing Property. Adv. Mater. Res..

[46] Qi J., White J.M., Belcher A.M., Masumoto Y. (2003). Optical spectroscopy of silicon nanowires. Chem. Phys. Lett..

[47] Koao L.F., Hone F.G., Dejene F.B. (2020). Synthesis and characterization of PbS nanowires doped with Tb3+ ions by using chemical bath deposition method. J. Nanostruct. Chem..

[48] Adu K.W., Gutiérrez H.R., Kim U.J., Sumanasekera G.U., Eklund P.C. (2005). Confined Phonons in Si Nanowires. Nano Lett..

[49] Nanda K.K., Sahu S.N., Soni R.K., Tripathy S. (1998). Raman spectroscopy of PbS nanocrystalline semiconductors. Phys. Rev. B.

[50] Baranov A.V., Bogdanov K.V., Ushakova E.V., Cherevkov S.A., Fedorov A.V., Tscharntke S. (2010). Comparative analysis of Raman spectra of PbS macro- and nanocrystals. Opt. Spectrosc..

[51] Prasad N., Karthikeyan B. (2018). Resonant and Off-Resonant Phonon Properties of Wurtzite ZnS: Effect of Morphology on Fröhlich Coupling and Phonon Lifetime. J. Phys. Chem. C.

[52] Habinshuti J., Kilian O., Cristini-Robbe O., Sashchiuk A., Addad A., Turrell S., Wirtz L. (2013). Anomalous quantum confinment of the longitudinal optical phonon mode in PbSe quantum dots. Phys. Rev. B.

[53] Viezbicke B.D., Patel S., Davis B.E., Birnie D.P. (2015). Evaluation of the Tauc method for optical absorption edge determination: ZnO thin films as a model system. Phys. Status solidi B.

[54] Moreels I., Lambert K., Smeets D., De Muynck D., Nollet T., Martins J.C., Hens Z. (2009). Size-Dependent Optical Properties of Colloidal PbS Quantum Dots. ACS Nano.

[55] Freddi S., Fabbri F., Cannizzaro A., Agati M., Dolbec R., Drera G., Castrucci P. (2020). High-temperature nitrogen annealing induced bonding states and photoluminescence changes in inductively coupled plasma torch synthesized silicon nanostructures. J. Appl. Phys..

[56] Ross G.G., Barba D., Martin F. (2008). Structure and luminescence of silicon nanocrystals embedded in SiO_2_. Int. J. Nanotechnol..

[57] Veena E., Bangera K.V., Shivakumar G.K. (2017). Effective role of thickness on structural, electrical and optical properties of lead sulphide thin films for photovoltaic applications. Mater. Sci. Eng. B.

